# Carbolic Salve Containing Calomel

**Published:** 1898-09

**Authors:** 


Carbolic Salve Containing Calomel. ℞.—White wax, 8 ounces; lard, 24 ounces; carbolic acid, 2½ ounces; calomel, 4 drachms; camphor, 1 drachm. M.—*Phar. Era.*

